# Bis{μ-5-(diethyl­amino)-2-[(2-oxidoeth­oxy)imino­meth­yl]phenolato}dicopper(II) acetone solvate

**DOI:** 10.1107/S1600536808024707

**Published:** 2008-08-06

**Authors:** Wen-Kui Dong, Xiao Chen, Xue-Ni He, Xiao-Lu Tang, Zhong-Wu Lv

**Affiliations:** a School of Chemical and Biological Engineering, Lanzhou Jiaotong University, Lanzhou 730070, People’s Republic of China

## Abstract

The title complex, [Cu_2_(C_13_H_18_N_2_O_3_)_2_]·C_3_H_6_O, has been synthesized by the reaction of copper(II) acetate monohydrate with 5,5′-bis­(diethyl­amino)-2,2′-[ethyl­enedioxy­bis(nitrilo­methyl­idyne)]diphenol, where one of the N—O bonds of the ligand was cleaved during the reaction. The complex mol­ecule has a μ-dialkoxo-bridged binuclear structure with both Cu^II^ centers exhibiting a square-planar coordination geometry.

## Related literature

For related literature, see: Bu *et al.* (1990 ▶); Dong *et al.* (2007*a* ▶,*b* ▶); Sun *et al.* (2008 ▶); Zhang *et al.* (2007 ▶).
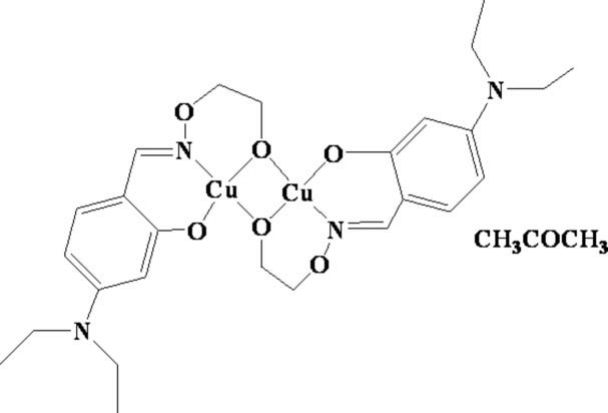


## Experimental

### 

#### Crystal data

[Cu_2_(C_13_H_18_N_2_O_3_)_2_]·C_3_H_6_O
*M* *_r_* = 685.75
Monoclinic, 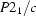

*a* = 20.633 (3) Å
*b* = 11.6045 (14) Å
*c* = 13.0738 (17) Å
β = 102.635 (2)°
*V* = 3054.6 (7) Å^3^
*Z* = 4
Mo *K*α radiation
μ = 1.44 mm^−1^
*T* = 298 (2) K
0.53 × 0.49 × 0.47 mm

#### Data collection

Bruker SMART 1000 CCD diffractometer
Absorption correction: multi-scan (*SADABS*; Sheldrick, 1996 ▶) *T*_min_ = 0.515, *T*_max_ = 0.550 (expected range = 0.475–0.507)
14726 measured reflections
5363 independent reflections
2967 reflections with *I* > 2σ(*I*)
*R*_int_ = 0.092

#### Refinement

*R*[*F* ^2^ > 2σ(*F* ^2^)] = 0.075
*wR*(*F* ^2^) = 0.229
*S* = 1.00
5363 reflections
385 parameters
H-atom parameters constrained
Δρ_max_ = 0.88 e Å^−3^
Δρ_min_ = −1.06 e Å^−3^

### 

Data collection: *SMART* (Siemens, 1996 ▶); cell refinement: *SAINT* (Siemens, 1996 ▶); data reduction: *SAINT*; program(s) used to solve structure: *SHELXS97* (Sheldrick, 2008 ▶); program(s) used to refine structure: *SHELXL97* (Sheldrick, 2008 ▶); molecular graphics: *SHELXTL* (Sheldrick, 2008 ▶); software used to prepare material for publication: *SHELXTL*.

## Supplementary Material

Crystal structure: contains datablocks global, I. DOI: 10.1107/S1600536808024707/gk2162sup1.cif

Structure factors: contains datablocks I. DOI: 10.1107/S1600536808024707/gk2162Isup2.hkl

Additional supplementary materials:  crystallographic information; 3D view; checkCIF report

## Figures and Tables

**Fig. 1.**
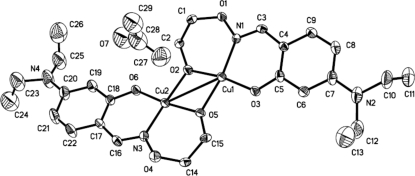
The molecular structure of the title compound with atom numbering scheme. Displacement ellipsoids for non-hydrogen atoms are drawn at the 30% probability level.

## supplementary crystallographic information

### Comment

Several works have been devoted to synthesize and characterize transition metal complexes bearing a salen-type bisoxime ligand or its derivatives (Dong *et al.*, 2007*a*; Dong *et al.*, 2007*b*).

The title compound has been synthesized by the reaction of copper(II) acetate monohydrate with a salen-type bisoxime ligand, 5,5'-di(*N*,*N*'-diethylamino)-2,2' -[ethylenedioxybis(nitrilomethylidyne)]diphenol (H_2_*L*^1^). The catalytic action of Cu^II^ ions resulted in unexpected cleavage of one of the N—O bonds in the ligand H_2_*L*^1^ (Bu *et al.*, 1990) giving a novel dialkoxo-bridged dinuclear complex with a Cu—O—Cu—O four-membered ring core, instead of the expected salen-type bisoxime Cu—N_2_O_2_ complex (Sun *et al.*, 2008).

The title molecule has µ-dialkoxo bridged binuclear structure with both Cu^II^ centers tetra-coordinated, where oxime nitrogen atom, phenoxo oxygen atom and two bridging alkoxo oxygen atoms act as donors. The Cu_2_O_2_ core is formed by two Cu^II^ ions and two bridging alkoxo oxygen atoms with Cu—Cu separation of 3.0051 (12) Å. The dihedral angle between the two planes, O2—Cu2—O5 and O2—Cu1—O5, is 8.80 (4)°.

### Experimental

5, 5'-Di(*N*,*N*'-diethylamino)-2,2'- [ethylenedioxybis(nitrilomethylidyne)]diphenol (H_2_*L*^1^) was synthesized according to previously reported procedure (Zhang *et al.*, 2007). A solution of copper(II) acetate monohydrate (20.0 mg, 0.1 mmol) in ethanol (15 ml) was added dropwise to a solution of H_2_*L*^1^ (44.3 mg, 0.1 mmol) in acetone (15 ml) at room temperature. The color of the mixing solution turned to brown immediately. The solution was stirred for 4 h at room temperature and then filtered. The filtrate was allowed to evaporate at room temperature for about three weeks and dark-brown prismatic single crystals suitable for X-ray crystallographic analysis were obtained. Anal. Calcd. for C_29_H_42_Cu_2_N_4_O_7_ {[Cu_2_(*L*^2^)_2_]C_3_H_6_O} (%): C, 50.79; H, 6.17; N, 8.17; Cu, 18.53. Found: C, 50.61; H, 6.19; N, 8.01; Cu, 18.29. IR: νC=N, 1614 cm^-1^ and νAr-O, 1235 cm^-1^.

### Refinement

H atoms were placed at calculated positions [C—H = 0.96 (CH_3_), 0.97 Å (CH_2_), 0.93 Å (CH)] and were included in the refinement in the riding model approximation, with *U*_iso_(H) = 1.2*U*_eq_(C).

### Crystal data

**Table:**

[Cu_2_(C_13_H_18_N_2_O_3_)_2_]·C_3_H_6_O	*F*_000_ = 1432
*M**_r_* = 685.75	*D*_x_ = 1.491 Mg m^−^^3^
Monoclinic, *P*2_1_/*c*	Mo *K*α radiation λ = 0.71073 Å
Hall symbol: -P 2ybc	Cell parameters from 2917 reflections
*a* = 20.633 (3) Å	θ = 2.4–22.9º
*b* = 11.6045 (14) Å	µ = 1.44 mm^−^^1^
*c* = 13.0738 (17) Å	*T* = 298 (2) K
β = 102.635 (2)º	Prismatic, dark-brown
*V* = 3054.6 (7) Å^3^	0.53 × 0.49 × 0.47 mm
*Z* = 4	

### Data collection

**Table:**

Bruker SMART 1000 CCD diffractometer	5363 independent reflections
Radiation source: fine-focus sealed tube	2967 reflections with *I* > 2σ(*I*)
Monochromator: graphite	*R*_int_ = 0.092
*T* = 298(2) K	θ_max_ = 25.0º
φ and ω scans	θ_min_ = 2.0º
Absorption correction: multi-scan(SADABS; Sheldrick, 1996)	*h* = −23→24
*T*_min_ = 0.515, *T*_max_ = 0.550	*k* = −13→9
14726 measured reflections	*l* = −15→15

### Refinement

**Table:**

Refinement on *F*^2^	Secondary atom site location: difference Fourier map
Least-squares matrix: full	Hydrogen site location: inferred from neighbouring sites
*R*[*F*^2^ > 2σ(*F*^2^)] = 0.075	H-atom parameters constrained
*wR*(*F*^2^) = 0.229	*w* = 1/[σ^2^(*F*_o_^2^) + (0.1245*P*)^2^] where *P* = (*F*_o_^2^ + 2*F*_c_^2^)/3
*S* = 1.00	(Δ/σ)_max_ = 0.001
5363 reflections	Δρ_max_ = 0.88 e Å^−^^3^
385 parameters	Δρ_min_ = −1.06 e Å^−^^3^
Primary atom site location: structure-invariant direct methods	Extinction correction: none

### Special details

**Table:**

Geometry. All e.s.d.'s (except the e.s.d. in the dihedral angle between two l.s. planes) are estimated using the full covariance matrix. The cell e.s.d.'s are taken into account individually in the estimation of e.s.d.'s in distances, angles and torsion angles; correlations between e.s.d.'s in cell parameters are only used when they are defined by crystal symmetry. An approximate (isotropic) treatment of cell e.s.d.'s is used for estimating e.s.d.'s involving l.s. planes.
Refinement. Refinement of *F*^2^ against ALL reflections. The weighted *R*-factor *wR* and goodness of fit *S* are based on *F*^2^, conventional *R*-factors *R* are based on *F*, with *F* set to zero for negative *F*^2^. The threshold expression of *F*^2^ > σ(*F*^2^) is used only for calculating *R*-factors(gt) *etc*. and is not relevant to the choice of reflections for refinement. *R*-factors based on *F*^2^ are statistically about twice as large as those based on *F*, and *R*- factors based on ALL data will be even larger.

### Fractional atomic coordinates and isotropic or equivalent isotropic displacement parameters (Å^2^)

**Table:**

	*x*	*y*	*z*	*U*_iso_*/*U*_eq_	
Cu1	0.50062 (4)	0.85967 (8)	0.08317 (7)	0.0325 (3)	
Cu2	0.40492 (4)	0.91965 (8)	0.21830 (7)	0.0350 (3)	
N1	0.4892 (3)	0.7990 (5)	−0.0554 (5)	0.0362 (15)	
N2	0.8064 (3)	0.8744 (7)	0.0240 (6)	0.061 (2)	
N3	0.4087 (3)	1.0305 (6)	0.3279 (5)	0.0419 (16)	
N4	0.0932 (4)	0.9250 (8)	0.2481 (7)	0.075 (3)	
O1	0.4275 (2)	0.7535 (4)	−0.1126 (4)	0.0392 (13)	
O2	0.4116 (2)	0.8328 (5)	0.0968 (4)	0.0412 (13)	
O3	0.5922 (2)	0.8847 (4)	0.0975 (4)	0.0398 (13)	
O4	0.4662 (2)	1.0969 (4)	0.3692 (4)	0.0447 (14)	
O5	0.4970 (2)	0.9318 (4)	0.2158 (4)	0.0395 (13)	
O6	0.3153 (2)	0.8842 (5)	0.2103 (4)	0.0456 (14)	
O7	0.1481 (6)	0.5560 (10)	0.0993 (9)	0.154 (4)	
C1	0.3732 (3)	0.8175 (7)	−0.0905 (6)	0.0398 (19)	
H1A	0.3337	0.7991	−0.1433	0.048*	
H1B	0.3822	0.8990	−0.0968	0.048*	
C2	0.3591 (3)	0.7965 (7)	0.0148 (6)	0.042 (2)	
H2A	0.3189	0.8373	0.0199	0.051*	
H2B	0.3513	0.7149	0.0226	0.051*	
C3	0.5347 (3)	0.7810 (6)	−0.1079 (6)	0.0386 (18)	
H3	0.5222	0.7487	−0.1744	0.046*	
C4	0.6025 (3)	0.8082 (7)	−0.0690 (6)	0.0389 (18)	
C5	0.6286 (4)	0.8589 (7)	0.0283 (6)	0.0413 (19)	
C6	0.6954 (4)	0.8808 (7)	0.0556 (7)	0.048 (2)	
H6	0.7128	0.9147	0.1203	0.058*	
C7	0.7386 (4)	0.8539 (8)	−0.0103 (7)	0.051 (2)	
C8	0.7128 (4)	0.8060 (7)	−0.1067 (7)	0.050 (2)	
H8	0.7405	0.7888	−0.1520	0.060*	
C9	0.6464 (3)	0.7839 (7)	−0.1356 (6)	0.044 (2)	
H9	0.6293	0.7518	−0.2012	0.053*	
C10	0.8505 (4)	0.8597 (8)	−0.0509 (8)	0.064 (3)	
H10A	0.8255	0.8780	−0.1209	0.077*	
H10B	0.8868	0.9143	−0.0334	0.077*	
C11	0.8781 (5)	0.7429 (9)	−0.0516 (8)	0.080 (3)	
H11A	0.9047	0.7255	0.0165	0.120*	
H11B	0.9051	0.7389	−0.1026	0.120*	
H11C	0.8425	0.6882	−0.0690	0.120*	
C12	0.8390 (5)	0.9014 (9)	0.1339 (8)	0.072 (3)	
H12A	0.8152	0.8672	0.1827	0.087*	
H12B	0.8847	0.8750	0.1506	0.087*	
C13	0.8354 (6)	1.0267 (10)	0.1358 (10)	0.097 (4)	
H13A	0.8504	1.0576	0.0769	0.146*	
H13B	0.8631	1.0550	0.1996	0.146*	
H13C	0.7903	1.0500	0.1321	0.146*	
C14	0.5245 (3)	1.0263 (7)	0.3815 (6)	0.0422 (19)	
H14A	0.5166	0.9544	0.4147	0.051*	
H14B	0.5611	1.0652	0.4280	0.051*	
C15	0.5443 (3)	0.9998 (7)	0.2812 (6)	0.0397 (19)	
H15A	0.5498	1.0713	0.2456	0.048*	
H15B	0.5866	0.9599	0.2960	0.048*	
C16	0.3585 (4)	1.0680 (7)	0.3631 (7)	0.049 (2)	
H16	0.3665	1.1265	0.4128	0.058*	
C17	0.2923 (4)	1.0267 (7)	0.3318 (7)	0.048 (2)	
C18	0.2740 (4)	0.9378 (8)	0.2589 (7)	0.051 (2)	
C19	0.2075 (4)	0.9048 (8)	0.2342 (7)	0.059 (3)	
H19	0.1954	0.8429	0.1889	0.071*	
C20	0.1578 (4)	0.9599 (9)	0.2739 (8)	0.065 (3)	
C21	0.1795 (5)	1.0508 (8)	0.3459 (8)	0.066 (3)	
H21	0.1487	1.0888	0.3761	0.079*	
C22	0.2412 (4)	1.0817 (8)	0.3705 (7)	0.057 (2)	
H22	0.2527	1.1436	0.4161	0.069*	
C23	0.0379 (5)	1.0076 (11)	0.2675 (10)	0.087 (3)	
H23A	0.0533	1.0868	0.2728	0.104*	
H23B	−0.0013	1.0020	0.2112	0.104*	
C24	0.0237 (6)	0.9686 (12)	0.3669 (10)	0.110 (4)	
H24A	0.0075	0.8908	0.3595	0.165*	
H24B	−0.0093	1.0177	0.3856	0.165*	
H24C	0.0636	0.9718	0.4208	0.165*	
C25	0.0727 (5)	0.8171 (10)	0.1932 (10)	0.083 (3)	
H25A	0.1089	0.7620	0.2083	0.100*	
H25B	0.0352	0.7848	0.2170	0.100*	
C26	0.0544 (6)	0.8385 (12)	0.0819 (10)	0.109 (4)	
H26A	0.0138	0.8818	0.0659	0.164*	
H26B	0.0482	0.7664	0.0450	0.164*	
H26C	0.0890	0.8815	0.0607	0.164*	
C27	0.2690 (8)	0.5540 (14)	0.1403 (14)	0.151 (6)	
H27A	0.2945	0.6170	0.1761	0.226*	
H27B	0.2914	0.5225	0.0894	0.226*	
H27C	0.2645	0.4954	0.1901	0.226*	
C28	0.2039 (10)	0.5947 (16)	0.0880 (15)	0.139 (6)	
C29	0.1968 (7)	0.6857 (14)	0.0057 (12)	0.135 (5)	
H29A	0.1514	0.6892	−0.0323	0.202*	
H29B	0.2249	0.6678	−0.0417	0.202*	
H29C	0.2095	0.7589	0.0382	0.202*	

### Atomic displacement parameters (Å^2^)

**Table:**

	*U*^11^	*U*^22^	*U*^33^	*U*^12^	*U*^13^	*U*^23^
Cu1	0.0317 (5)	0.0379 (6)	0.0287 (5)	−0.0031 (4)	0.0086 (4)	−0.0046 (4)
Cu2	0.0319 (5)	0.0410 (6)	0.0332 (6)	−0.0024 (4)	0.0093 (4)	−0.0071 (4)
N1	0.030 (3)	0.043 (4)	0.036 (4)	−0.004 (3)	0.010 (3)	−0.005 (3)
N2	0.047 (4)	0.071 (5)	0.067 (6)	0.000 (4)	0.018 (4)	−0.004 (4)
N3	0.036 (3)	0.045 (4)	0.047 (4)	−0.005 (3)	0.014 (3)	−0.010 (3)
N4	0.057 (5)	0.081 (6)	0.090 (7)	−0.005 (5)	0.025 (5)	−0.023 (5)
O1	0.034 (3)	0.046 (3)	0.038 (3)	−0.003 (2)	0.009 (2)	−0.014 (3)
O2	0.034 (3)	0.052 (3)	0.039 (3)	−0.006 (2)	0.012 (2)	−0.011 (3)
O3	0.034 (3)	0.047 (3)	0.041 (3)	−0.007 (2)	0.013 (2)	−0.010 (3)
O4	0.042 (3)	0.042 (3)	0.050 (4)	−0.008 (3)	0.011 (3)	−0.011 (3)
O5	0.035 (3)	0.048 (3)	0.037 (3)	−0.008 (2)	0.013 (2)	−0.011 (3)
O6	0.038 (3)	0.051 (3)	0.050 (4)	−0.004 (3)	0.015 (3)	−0.017 (3)
O7	0.137 (9)	0.164 (11)	0.162 (11)	−0.028 (8)	0.036 (8)	−0.006 (8)
C1	0.033 (4)	0.046 (5)	0.038 (5)	−0.004 (4)	0.005 (3)	−0.007 (4)
C2	0.035 (4)	0.051 (5)	0.041 (5)	−0.004 (4)	0.009 (3)	−0.011 (4)
C3	0.036 (4)	0.042 (5)	0.038 (5)	−0.003 (4)	0.010 (3)	−0.003 (4)
C4	0.036 (4)	0.043 (5)	0.041 (5)	−0.001 (4)	0.014 (3)	−0.004 (4)
C5	0.036 (4)	0.045 (5)	0.046 (5)	−0.001 (4)	0.015 (4)	−0.005 (4)
C6	0.039 (5)	0.055 (5)	0.052 (5)	−0.005 (4)	0.015 (4)	−0.004 (4)
C7	0.041 (5)	0.057 (6)	0.059 (6)	−0.003 (4)	0.017 (4)	−0.004 (5)
C8	0.042 (5)	0.058 (6)	0.055 (6)	0.000 (4)	0.019 (4)	−0.003 (5)
C9	0.041 (4)	0.048 (5)	0.045 (5)	0.001 (4)	0.014 (4)	−0.003 (4)
C10	0.049 (5)	0.074 (7)	0.072 (7)	−0.001 (5)	0.020 (5)	−0.002 (6)
C11	0.066 (6)	0.085 (8)	0.092 (8)	0.011 (6)	0.026 (6)	0.006 (7)
C12	0.053 (6)	0.087 (8)	0.078 (8)	−0.004 (6)	0.017 (5)	−0.007 (6)
C13	0.090 (8)	0.088 (9)	0.111 (11)	−0.004 (7)	0.017 (7)	−0.006 (8)
C14	0.038 (4)	0.044 (5)	0.043 (5)	−0.008 (4)	0.006 (4)	−0.008 (4)
C15	0.034 (4)	0.044 (5)	0.040 (5)	−0.010 (4)	0.008 (3)	−0.009 (4)
C16	0.045 (5)	0.050 (5)	0.053 (6)	−0.002 (4)	0.017 (4)	−0.014 (4)
C17	0.043 (5)	0.052 (5)	0.054 (6)	−0.005 (4)	0.022 (4)	−0.016 (5)
C18	0.044 (5)	0.058 (6)	0.058 (6)	−0.003 (4)	0.023 (4)	−0.014 (5)
C19	0.049 (5)	0.064 (6)	0.069 (7)	−0.005 (5)	0.024 (5)	−0.017 (5)
C20	0.053 (6)	0.071 (7)	0.076 (7)	−0.006 (5)	0.023 (5)	−0.018 (6)
C21	0.055 (6)	0.071 (7)	0.077 (7)	−0.003 (5)	0.027 (5)	−0.020 (6)
C22	0.050 (5)	0.060 (6)	0.065 (6)	−0.004 (5)	0.023 (5)	−0.019 (5)
C23	0.069 (7)	0.096 (9)	0.098 (10)	−0.007 (6)	0.025 (6)	−0.018 (7)
C24	0.097 (9)	0.115 (11)	0.116 (12)	−0.014 (8)	0.019 (8)	−0.015 (9)
C25	0.063 (7)	0.092 (9)	0.100 (10)	−0.008 (6)	0.027 (6)	−0.021 (8)
C26	0.097 (9)	0.118 (11)	0.108 (12)	−0.006 (8)	0.013 (8)	−0.005 (9)
C27	0.123 (13)	0.170 (18)	0.157 (17)	−0.019 (12)	0.025 (12)	0.007 (13)
C28	0.128 (14)	0.151 (16)	0.145 (16)	−0.031 (13)	0.047 (13)	0.010 (12)
C29	0.125 (12)	0.144 (14)	0.135 (14)	−0.020 (11)	0.028 (10)	−0.008 (12)

### Geometric parameters (Å, °)

**Table:**

Cu1—O3	1.880 (5)	C11—H11A	0.9600
Cu1—O2	1.909 (5)	C11—H11B	0.9600
Cu1—N1	1.910 (6)	C11—H11C	0.9600
Cu1—O5	1.942 (5)	C12—C13	1.456 (14)
Cu1—Cu2	3.0051 (12)	C12—H12A	0.9700
Cu2—O6	1.874 (5)	C12—H12B	0.9700
Cu2—O2	1.911 (5)	C13—H13A	0.9600
Cu2—O5	1.912 (5)	C13—H13B	0.9600
Cu2—N3	1.914 (6)	C13—H13C	0.9600
N1—C3	1.295 (8)	C14—C15	1.488 (10)
N1—O1	1.428 (7)	C14—H14A	0.9700
N2—C7	1.394 (10)	C14—H14B	0.9700
N2—C12	1.480 (12)	C15—H15A	0.9700
N2—C10	1.485 (11)	C15—H15B	0.9700
N3—C16	1.297 (9)	C16—C17	1.421 (11)
N3—O4	1.419 (7)	C16—H16	0.9300
N4—C20	1.363 (11)	C17—C18	1.400 (11)
N4—C25	1.459 (13)	C17—C22	1.416 (10)
N4—C23	1.552 (12)	C18—C19	1.392 (11)
O1—C1	1.425 (8)	C19—C20	1.401 (11)
O2—C2	1.412 (8)	C19—H19	0.9300
O3—C5	1.330 (8)	C20—C21	1.419 (13)
O4—C14	1.435 (8)	C21—C22	1.295 (11)
O5—C15	1.393 (8)	C21—H21	0.9300
O6—C18	1.324 (8)	C22—H22	0.9300
O7—C28	1.275 (16)	C23—C24	1.465 (14)
C1—C2	1.488 (10)	C23—H23A	0.9700
C1—H1A	0.9700	C23—H23B	0.9700
C1—H1B	0.9700	C24—H24A	0.9600
C2—H2A	0.9700	C24—H24B	0.9600
C2—H2B	0.9700	C24—H24C	0.9600
C3—C4	1.417 (10)	C25—C26	1.442 (15)
C3—H3	0.9300	C25—H25A	0.9700
C4—C5	1.398 (11)	C25—H25B	0.9700
C4—C9	1.415 (10)	C26—H26A	0.9600
C5—C6	1.371 (10)	C26—H26B	0.9600
C6—C7	1.403 (11)	C26—H26C	0.9600
C6—H6	0.9300	C27—C28	1.45 (2)
C7—C8	1.374 (12)	C27—H27A	0.9600
C8—C9	1.365 (10)	C27—H27B	0.9600
C8—H8	0.9300	C27—H27C	0.9600
C9—H9	0.9300	C28—C29	1.49 (2)
C10—C11	1.470 (13)	C29—H29A	0.9600
C10—H10A	0.9700	C29—H29B	0.9600
C10—H10B	0.9700	C29—H29C	0.9600
			
O3—Cu1—O2	169.2 (2)	H11B—C11—H11C	109.5
O3—Cu1—N1	93.7 (2)	C13—C12—N2	102.3 (9)
O2—Cu1—N1	96.1 (2)	C13—C12—H12A	111.3
O3—Cu1—O5	94.5 (2)	N2—C12—H12A	111.3
O2—Cu1—O5	76.2 (2)	C13—C12—H12B	111.3
N1—Cu1—O5	170.1 (2)	N2—C12—H12B	111.3
O3—Cu1—Cu2	132.92 (15)	H12A—C12—H12B	109.2
O2—Cu1—Cu2	38.15 (15)	C12—C13—H13A	109.5
N1—Cu1—Cu2	133.02 (17)	C12—C13—H13B	109.5
O5—Cu1—Cu2	38.40 (13)	H13A—C13—H13B	109.5
O6—Cu2—O2	95.0 (2)	C12—C13—H13C	109.5
O6—Cu2—O5	170.6 (2)	H13A—C13—H13C	109.5
O2—Cu2—O5	76.8 (2)	H13B—C13—H13C	109.5
O6—Cu2—N3	93.9 (2)	O4—C14—C15	113.7 (6)
O2—Cu2—N3	167.9 (2)	O4—C14—H14A	108.8
O5—Cu2—N3	94.9 (2)	C15—C14—H14A	108.8
O6—Cu2—Cu1	133.10 (16)	O4—C14—H14B	108.8
O2—Cu2—Cu1	38.11 (14)	C15—C14—H14B	108.8
O5—Cu2—Cu1	39.11 (14)	H14A—C14—H14B	107.7
N3—Cu2—Cu1	132.15 (18)	O5—C15—C14	111.6 (6)
C3—N1—O1	109.2 (6)	O5—C15—H15A	109.3
C3—N1—Cu1	127.5 (5)	C14—C15—H15A	109.3
O1—N1—Cu1	123.0 (4)	O5—C15—H15B	109.3
C7—N2—C12	124.0 (7)	C14—C15—H15B	109.3
C7—N2—C10	119.0 (8)	H15A—C15—H15B	108.0
C12—N2—C10	116.8 (7)	N3—C16—C17	125.3 (8)
C16—N3—O4	110.4 (6)	N3—C16—H16	117.4
C16—N3—Cu2	125.9 (6)	C17—C16—H16	117.4
O4—N3—Cu2	122.9 (4)	C18—C17—C22	117.7 (7)
C20—N4—C25	122.6 (8)	C18—C17—C16	122.9 (7)
C20—N4—C23	119.3 (8)	C22—C17—C16	119.3 (8)
C25—N4—C23	117.8 (8)	O6—C18—C19	117.7 (8)
C1—O1—N1	110.6 (5)	O6—C18—C17	124.5 (7)
C2—O2—Cu1	124.9 (4)	C19—C18—C17	117.8 (7)
C2—O2—Cu2	127.3 (4)	C18—C19—C20	123.6 (9)
Cu1—O2—Cu2	103.7 (2)	C18—C19—H19	118.2
C5—O3—Cu1	127.0 (5)	C20—C19—H19	118.2
N3—O4—C14	110.0 (5)	N4—C20—C19	121.9 (9)
C15—O5—Cu2	126.6 (4)	N4—C20—C21	122.3 (8)
C15—O5—Cu1	129.3 (4)	C19—C20—C21	115.8 (8)
Cu2—O5—Cu1	102.5 (2)	C22—C21—C20	121.4 (9)
C18—O6—Cu2	126.9 (5)	C22—C21—H21	119.3
O1—C1—C2	115.1 (6)	C20—C21—H21	119.3
O1—C1—H1A	108.5	C21—C22—C17	123.7 (9)
C2—C1—H1A	108.5	C21—C22—H22	118.2
O1—C1—H1B	108.5	C17—C22—H22	118.2
C2—C1—H1B	108.5	C24—C23—N4	104.5 (10)
H1A—C1—H1B	107.5	C24—C23—H23A	110.8
O2—C2—C1	112.4 (6)	N4—C23—H23A	110.8
O2—C2—H2A	109.1	C24—C23—H23B	110.8
C1—C2—H2A	109.1	N4—C23—H23B	110.8
O2—C2—H2B	109.1	H23A—C23—H23B	108.9
C1—C2—H2B	109.1	C23—C24—H24A	109.5
H2A—C2—H2B	107.9	C23—C24—H24B	109.5
N1—C3—C4	123.2 (7)	H24A—C24—H24B	109.5
N1—C3—H3	118.4	C23—C24—H24C	109.5
C4—C3—H3	118.4	H24A—C24—H24C	109.5
C5—C4—C9	118.3 (7)	H24B—C24—H24C	109.5
C5—C4—C3	125.0 (7)	C26—C25—N4	109.4 (11)
C9—C4—C3	116.7 (7)	C26—C25—H25A	109.8
O3—C5—C6	117.8 (7)	N4—C25—H25A	109.8
O3—C5—C4	123.5 (7)	C26—C25—H25B	109.8
C6—C5—C4	118.7 (7)	N4—C25—H25B	109.8
C5—C6—C7	122.4 (8)	H25A—C25—H25B	108.2
C5—C6—H6	118.8	C25—C26—H26A	109.5
C7—C6—H6	118.8	C25—C26—H26B	109.5
C8—C7—N2	121.6 (8)	H26A—C26—H26B	109.5
C8—C7—C6	118.9 (8)	C25—C26—H26C	109.5
N2—C7—C6	119.5 (8)	H26A—C26—H26C	109.5
C9—C8—C7	119.6 (8)	H26B—C26—H26C	109.5
C9—C8—H8	120.2	C28—C27—H27A	109.5
C7—C8—H8	120.2	C28—C27—H27B	109.5
C8—C9—C4	122.1 (8)	H27A—C27—H27B	109.5
C8—C9—H9	119.0	C28—C27—H27C	109.5
C4—C9—H9	119.0	H27A—C27—H27C	109.5
C11—C10—N2	113.8 (8)	H27B—C27—H27C	109.5
C11—C10—H10A	108.8	O7—C28—C27	126.8 (18)
N2—C10—H10A	108.8	O7—C28—C29	112.6 (17)
C11—C10—H10B	108.8	C27—C28—C29	120.5 (16)
N2—C10—H10B	108.8	C28—C29—H29A	109.5
H10A—C10—H10B	107.7	C28—C29—H29B	109.5
C10—C11—H11A	109.5	H29A—C29—H29B	109.5
C10—C11—H11B	109.5	C28—C29—H29C	109.5
H11A—C11—H11B	109.5	H29A—C29—H29C	109.5
C10—C11—H11C	109.5	H29B—C29—H29C	109.5
H11A—C11—H11C	109.5		
			
O3—Cu1—Cu2—O6	−173.8 (3)	N1—O1—C1—C2	−73.6 (8)
O2—Cu1—Cu2—O6	−2.9 (3)	Cu1—O2—C2—C1	−18.9 (9)
N1—Cu1—Cu2—O6	14.9 (4)	Cu2—O2—C2—C1	134.5 (6)
O5—Cu1—Cu2—O6	−172.3 (3)	O1—C1—C2—O2	64.6 (9)
O3—Cu1—Cu2—O2	−170.9 (3)	O1—N1—C3—C4	−175.2 (7)
N1—Cu1—Cu2—O2	17.8 (4)	Cu1—N1—C3—C4	−1.9 (11)
O5—Cu1—Cu2—O2	−169.3 (4)	N1—C3—C4—C5	−1.2 (13)
O3—Cu1—Cu2—O5	−1.6 (3)	N1—C3—C4—C9	−179.8 (7)
O2—Cu1—Cu2—O5	169.3 (4)	Cu1—O3—C5—C6	−177.8 (5)
N1—Cu1—Cu2—O5	−172.8 (4)	Cu1—O3—C5—C4	0.5 (11)
O3—Cu1—Cu2—N3	19.7 (4)	C9—C4—C5—O3	−179.4 (7)
O2—Cu1—Cu2—N3	−169.3 (4)	C3—C4—C5—O3	2.0 (13)
N1—Cu1—Cu2—N3	−151.5 (4)	C9—C4—C5—C6	−1.2 (12)
O5—Cu1—Cu2—N3	21.3 (4)	C3—C4—C5—C6	−179.8 (8)
O3—Cu1—N1—C3	3.2 (7)	O3—C5—C6—C7	178.2 (7)
O2—Cu1—N1—C3	−172.3 (7)	C4—C5—C6—C7	−0.2 (13)
Cu2—Cu1—N1—C3	176.8 (5)	C12—N2—C7—C8	−166.2 (9)
O3—Cu1—N1—O1	175.6 (5)	C10—N2—C7—C8	8.8 (13)
O2—Cu1—N1—O1	0.1 (5)	C12—N2—C7—C6	12.7 (13)
Cu2—Cu1—N1—O1	−10.8 (7)	C10—N2—C7—C6	−172.3 (8)
O6—Cu2—N3—C16	−8.1 (7)	C5—C6—C7—C8	1.4 (14)
O2—Cu2—N3—C16	129.1 (11)	C5—C6—C7—N2	−177.5 (8)
O5—Cu2—N3—C16	175.3 (7)	N2—C7—C8—C9	177.8 (8)
Cu1—Cu2—N3—C16	162.0 (6)	C6—C7—C8—C9	−1.1 (13)
O6—Cu2—N3—O4	−177.0 (5)	C7—C8—C9—C4	−0.3 (13)
O2—Cu2—N3—O4	−39.8 (15)	C5—C4—C9—C8	1.5 (12)
O5—Cu2—N3—O4	6.4 (6)	C3—C4—C9—C8	−179.8 (7)
Cu1—Cu2—N3—O4	−6.9 (7)	C7—N2—C10—C11	−90.6 (11)
C3—N1—O1—C1	−150.4 (6)	C12—N2—C10—C11	84.7 (11)
Cu1—N1—O1—C1	36.0 (7)	C7—N2—C12—C13	−90.3 (10)
O3—Cu1—O2—C2	−163.5 (10)	C10—N2—C12—C13	94.6 (10)
N1—Cu1—O2—C2	−8.5 (6)	N3—O4—C14—C15	75.3 (7)
O5—Cu1—O2—C2	165.3 (6)	Cu2—O5—C15—C14	23.7 (9)
Cu2—Cu1—O2—C2	158.5 (7)	Cu1—O5—C15—C14	−173.3 (5)
O3—Cu1—O2—Cu2	38.0 (13)	O4—C14—C15—O5	−65.7 (9)
N1—Cu1—O2—Cu2	−167.0 (3)	O4—N3—C16—C17	175.1 (8)
O5—Cu1—O2—Cu2	6.8 (2)	Cu2—N3—C16—C17	5.1 (13)
O6—Cu2—O2—C2	20.1 (6)	N3—C16—C17—C18	1.0 (15)
O5—Cu2—O2—C2	−164.7 (6)	N3—C16—C17—C22	−175.1 (8)
N3—Cu2—O2—C2	−117.0 (12)	Cu2—O6—C18—C19	173.8 (6)
Cu1—Cu2—O2—C2	−157.8 (7)	Cu2—O6—C18—C17	−5.0 (13)
O6—Cu2—O2—Cu1	177.9 (3)	C22—C17—C18—O6	175.0 (8)
O5—Cu2—O2—Cu1	−6.9 (2)	C16—C17—C18—O6	−1.2 (15)
N3—Cu2—O2—Cu1	40.8 (13)	C22—C17—C18—C19	−3.8 (13)
O2—Cu1—O3—C5	152.7 (11)	C16—C17—C18—C19	−179.9 (9)
N1—Cu1—O3—C5	−2.4 (6)	O6—C18—C19—C20	−175.2 (9)
O5—Cu1—O3—C5	−177.0 (6)	C17—C18—C19—C20	3.6 (15)
Cu2—Cu1—O3—C5	−176.0 (5)	C25—N4—C20—C19	11.0 (16)
C16—N3—O4—C14	148.0 (7)	C23—N4—C20—C19	−162.8 (10)
Cu2—N3—O4—C14	−41.6 (7)	C25—N4—C20—C21	−166.1 (11)
O2—Cu2—O5—C15	173.3 (6)	C23—N4—C20—C21	20.1 (15)
N3—Cu2—O5—C15	2.3 (6)	C18—C19—C20—N4	−179.8 (9)
Cu1—Cu2—O5—C15	166.6 (7)	C18—C19—C20—C21	−2.5 (15)
O2—Cu2—O5—Cu1	6.7 (2)	N4—C20—C21—C22	179.1 (10)
N3—Cu2—O5—Cu1	−164.3 (3)	C19—C20—C21—C22	1.8 (15)
O3—Cu1—O5—C15	12.8 (6)	C20—C21—C22—C17	−2.3 (16)
O2—Cu1—O5—C15	−172.8 (7)	C18—C17—C22—C21	3.3 (15)
Cu2—Cu1—O5—C15	−166.1 (8)	C16—C17—C22—C21	179.6 (9)
O3—Cu1—O5—Cu2	178.8 (2)	C20—N4—C23—C24	−98.5 (11)
O2—Cu1—O5—Cu2	−6.8 (2)	C25—N4—C23—C24	87.4 (12)
O2—Cu2—O6—C18	−163.7 (7)	C20—N4—C25—C26	−93.3 (12)
N3—Cu2—O6—C18	8.0 (7)	C23—N4—C25—C26	80.6 (12)
Cu1—Cu2—O6—C18	−161.9 (6)		

## References

[bb1] Bu, X. R., You, X. Z. & Meng, Q. J. (1990). *Comments Inorg. Chem.* **9**, 221–244.

[bb2] Dong, W. K., Chen, X., Wang, S. J., He, X. N., Wu, H. L. & Yu, T. Z. (2007*a*). *Synth. React. Inorg. Met. Org. Nano-Met. Chem.* **37**, 229–233.

[bb3] Dong, W. K., Duan, J. G. & Liu, G. L. (2007*b*). *Transition Met. Chem.* **32**, 702–705.

[bb4] Sheldrick, G. M. (1996). *SADABS* University of Göttingen, Germany.

[bb5] Sheldrick, G. M. (2008). *Acta Cryst.* A**64**, 112–122. 10.1107/S0108767307043930 18156677

[bb6] Siemens (1996). *SMART* and *SAINT* Siemens Analytical X-ray Instruments Inc., Madison, Wisconsin, USA.

[bb7] Sun, Y.-X., Gao, S.-X., Shi, J.-Y. & Dong, W.-K. (2008). *Acta Cryst.* E**64**, m226. 10.1107/S1600536807061752 PMC2924182 21200572

[bb8] Zhang, Y.-P., Chen, X., Shi, J.-Y., Xu, L. & Dong, W.-K. (2007). *Acta Cryst.* E**63**, o3852.

